# Patterns of tobacco product use and substance misuse among adolescents in the United States

**DOI:** 10.1016/j.pmedr.2023.102207

**Published:** 2023-04-15

**Authors:** John Erhabor, Ellen Boakye, Ngozi Osuji, Olufunmilayo Obisesan, Albert D. Osei, Hassan Mirbolouk, Andrew C. Stokes, Omar Dzaye, Omar El-Shahawy, Carlos J. Rodriguez, Glenn A. Hirsch, Emelia J. Benjamin, Andrew P. DeFilippis, Rose Marie Robertson, Aruni Bhatnagar, Michael J. Blaha

**Affiliations:** a Johns Hopkins Ciccarone Center for Prevention of Cardiovascular Disease, Baltimore, MD, USA; b American Heart Association Tobacco Regulation and Addiction Center, Dallas, TX, USA; c Department of Medicine, MedStar Union Memorial Hospital, Baltimore, MD, USA; d Department of Internal Medicine, Yale School of Medicine, New Haven, CT, USA; e Department of Global Health, Boston University School of Public Health, Boston, MA, USA; f Department of Population Health, New York University School of Medicine, New York, NY, USA; g Albert Einstein College of Medicine, Bronx, New York, NY, USA; h Division of Cardiology, Department of Medicine, National Jewish Health, Denver, CO, USA; i Cardiovascular Medicine, Boston Medical Center, Boston University School of Medicine, Boston, MA, USA; j Department of Epidemiology, Boston University School of Public Health, Boston, MA, USA; k Department of Medicine, Vanderbilt University Medical Center, Nashville, TN, USA; l University of Louisville School of Medicine, Louisville, KY, USA

**Keywords:** Youth, E-cigarette, Substance misuse, Poly tobacco use, High school students

## Abstract

Among adolescents, sole use is the most common pattern of e-cigarette use. However, concurrent use of e-cigarettes with other tobacco products is not uncommon and may be associated with high-risk behaviors. We used data from 12,767 participants in the 2019 Youth Risk Behavior Survey to examine the patterns of tobacco product use among youth in the US. First, we examined the prevalence of e-cigarette-specific patterns of tobacco use (nonuse[no tobacco product use], sole use[sole e-cigarette use], dual-use[e-cigarette and one other tobacco product], and poly use[e-cigarette and two or more other tobacco products]). Then, using multivariable Poisson regression, we assessed how the tobacco use patterns were associated with the misuse of nine substances of abuse (alcohol, marijuana, cocaine, ecstasy, hallucinogens, heroin, inhalants, injectables, and methamphetamines).

62.9% of youth reported nonuse of any tobacco product. The weighted prevalence of sole e-cigarette use, dual use, and poly use was 23.2%, 4.2%, and 3.3%, respectively. Across all the substances explored, the prevalence was highest among poly users, followed by dual users, sole users, and non-users. Compared to non-users, sole, dual, and poly users had 7.8(95 %CI:6.1–10.0), 14.3(95 %CI:10.8–18.8), and 19.7(95 %CI:15.0–25.9) times higher adjusted prevalence of reporting past-30-day binge drinking, after adjusting for age, sex, race/ethnicity, sexual orientation, and depressive symptoms. This pattern was seen across all the different substances explored. These findings highlight the high prevalence of substance misuse among youth who use tobacco products and the need to educate and counsel on substances of abuse among this population, particularly among poly-tobacco users.

## Highlights

• The use of e-cigarettes in combination with other tobacco products among youth is not uncommon.
• Sole e-cigarette, dual and poly users of tobacco products had a higher prevalence of substance misuse than nonusers.
• Dual and poly users were more likely to engage in substance misuse than sole e-cigarette users.
• These findings highlight the clustering of tobacco use with substance misuse and emphasize the need for comprehensive efforts to address youth high-risk behaviors.

## 1 Introduction

E-cigarettes were first introduced into the United States (US) market in the mid-2000s, and by 2014, they had become the most used tobacco product among youth in the US, surpassing combustible cigarette use. (U.S. Department of Health and Human Services, 2016, Wang et al., 2018) More recent data have shown that there has been a decline in youth e-cigarette use. (Wang et al., 2021, Park-Lee et al., 2021) Despite the recent decline in e-cigarette use among adolescents and young adults, their use among this population remains of public health concern. (Wang et al., 2021, Park-Lee et al., 2021, Boakye et al., 2022) The possible reasons behind the reported decline in e-cigarette use among adolescents and young adults might be a result of tobacco regulatory policies that restrict youth access to e-cigarettes (e.g., Tobacco-21 Legislation), (Tobacco 21 | FDA, 2022) the ban on flavored e-cigarettes, (Ali et al., 2022) or the increase in the perceived harm of nicotine vaping. (Miech et al., 2021) Despite the decline in the prevalence of e-cigarette use among adolescents, their use is still widespread, with approximately 11.3% of high school students and 2.8% of middle school students reporting past-30-day e-cigarette use in 2021. (Park-Lee et al., 2021).

Most e-cigarettes contain nicotine, which is highly addictive and can harm the developing brain of adolescents. (Yuan et al., 2015, Goriounova and Mansvelder, 2012b) Also, the use of e-cigarettes in adolescents may serve as a gateway to the use of other tobacco products, although evidence is mixed as others postulate that such use patterns/transitions may be due to common underlying risk factors. (McCabe et al., 2018, Ren and Lotfipour, 2019, O’Brien et al., 2021, Berry et al., 2019) Additionally, e-cigarettes are typically the first and the most common tobacco products used by adolescents and are the most frequently used product in combination with other tobacco products. (Park-Lee et al., 2021, Berry et al., 2019, Gentzke et al., 2019) the use of multiple tobacco products is an emerging and concerning pattern of tobacco use among adolescents. In 2022, 5% of high school students reported past-30-day use of two or more tobacco products. (Park-Lee et al., 2022).

The use of e-cigarettes has been shown to be associated with substance use behaviors, (Demissie et al., 2017) and other high-risk behaviors. (Demissie et al., 2017, Jacobs et al., 2021) Given the rising prevalence of multiple tobacco product use among youth, (Jebai et al., 2022) it is crucial to examine the patterns of tobacco use among youth, particularly the e-cigarette-specific patterns of tobacco product use, and how they are associated with other high-risk behaviors such as substance misuse. To assess the prevalence of these patterns and their association with substance misuse, we used data from one of the largest nationally representative surveys of high school students in the US, the Youth Risk Behavior Surveillance System (YRBSS Overview, 2023). We hypothesized that adolescents who use e-cigarettes either exclusively or in combination with other tobacco products will have a higher prevalence of substance misuse compared to those who did not use any tobacco product.

## 2 Methods

### 2.1 Study sample

The national YRBSS is a biennial school-based survey that tracks high-risk health behaviors among high school students. The survey employs a three-stage cluster sample design to obtain a nationally representative sample of public and private school students in grades 9–12 across the US. (Overview, 2023) Students’ involvement is confidential and voluntary, and parental consent was obtained. The data obtained are self-reported and anonymous. The YRBSS school response rate in 2019 was 75%, and the student response rate was 80% resulting in an overall response rate of 60%. (Underwood et al., 2020) Of the 13,677 survey respondents in 2019, we included 12,767 who had information on the explored tobacco products (e-cigarettes, combustible cigarettes, cigars, and smokeless tobacco).

The Centers for Disease Control and Prevention's institutional review board authorized the national YRBS. Our work was exempt from institutional review board approval since we used de-identified, publicly accessible YRBSS data. A detailed description of the methodology used for the YRBSS has been published previously. (Underwood et al., 2020) The YRBSS survey questions have undergone test-retest analysis and have been demonstrated to have good reliability. (Underwood et al., 2020, Brener et al., 2003, Brener et al., 2002).

### 2.2 Assessment of tobacco use

The patterns of tobacco use were based on past-30-day use of the products and included nonuse (no tobacco product use), sole use (exclusive e-cigarette use), dual-use (e-cigarette use and one other tobacco product), poly use (e-cigarette use and two or three other tobacco products), and “others”. “Others” included the patterns of tobacco use other than the four categories of interest. This included sole, dual, and polyuse of other non-e-cigarette tobacco products. The following questions were used to assess e-cigarette use: “During the past 30 days, on how many days did you use an electronic vapor product?”; combustible cigarette use: “During the 30 days, on how many days did you smoke cigarettes?”; cigar use: “During the past 30 days, on how many days did you smoke cigars, cigarillos, or little cigars?”; and smokeless tobacco use: “During the past 30 days, on how many days did you use chewing tobacco, snuff, dip, snus, or dissolvable tobacco products, such as Copenhagen, Grizzly, Skoal, Camel Snus?”. Participants who reported using any of these products at least once in the past 30 days were considered current users of the product.

### 2.3 Substance misuse

The dependent variables of interest were the misuse of nine different substances of abuse. These included past-30-day binge drinking, past-30-day marijuana use, and lifetime use of cocaine, ecstasy, hallucinogens, heroin, inhalants, injectables, and methamphetamines. The exact questions used to assess substance misuse are presented in Supplementary Table S1.

### 2.4 Other measures

Sociodemographic variables included in this study were age, sex (female; male), race/ethnicity (American Indian/Alaskan Native/Native Hawaiian/Pacific Islander; Asian; African American; White; Hispanic; Multi-racial), grade (9th, 10th, 11th, and 12th), and sexual orientation (Heterosexual; Gay or Lesbian; Bisexual; not sure). Depression, used as a proxy for mental health, was assessed with the question, “During the past 12 months, did you ever feel so sad or hopeless almost every day for two weeks or more in a row that you stopped doing some usual activities?”

### 2.5 Statistical analysis

The age, sex, race/ethnicity, grade, and sexual orientation compositions of the study population were assessed first overall and then by the various tobacco use patterns of interest. We also estimated the weighted prevalence of the various substances first among the entire study population, then by the different tobacco use patterns. Multivariable Poisson regression models were used to estimate the adjusted prevalence ratios (aPRs) and 95% confidence intervals (CIs) of the association between each substance of abuse and the different e-cigarette use patterns. We further stratified the sole e-cigarette use group by frequency of use (<20 days and ≥20 days in the past month). First, we adjusted for age, sex, race/ethnicity, and sexual orientation (Model 1). Then, due to the frequent co-occurrence of mental health with both tobacco and substance use, we additionally adjusted for symptoms of depression in Model 2. We did not additionally adjust for grade in our models due to its high correlation with age (Pearson Correlation Coefficient = 0.87). In our primary analysis, we used non-users as the reference group. In an additional analysis, we used sole e-cigarette users as the reference.

The survey command “svy” was used to account for the complex survey design used by the YRBS. All analyses were carried out on weighted data using STATA version 17 (StataCorp, College Station, Tx). A 2-sided alpha (α) level of < 0.05 was used to determine statistical significance.

## 3 Results

Among the 12,767 participants, the weighted proportion of persons ≥18 years was 13.4%. Females constituted 49.5%, while the proportion of African American persons and White persons was 11.9% and 52.2%, respectively. Approximately 62.9% of participants reported nonuse of any tobacco product. The weighted prevalence of sole e-cigarette use, dual use, and poly use was 23.2%, 4.2%, and 3.3%, respectively. Compared to non-users and sole users, dual and poly users had a higher proportion of persons aged ≥18 years (10.9% vs. 15.7% vs. 20.2% vs. 26.0%), males (50.8% vs. 45.0% vs. 59.6% vs. 67.4%), and persons who identified as bisexual (8.1% vs. 8.7% vs. 10.2% vs. 12.5%) (Table 1).

**Table 1:** Sociodemographic Characteristics of Study Population based on Patterns of E-cigarette Use, 2019 Youth Risk Behavior Surveillance System, United States.

Sociodemographic Characteristics	Total N = 12,767 (Weighted %)	Nonuse N = 8,127 (Weighted %)	Sole Use N = 2,893 (Weighted %)	Dual Use N = 567 (Weighted %)	Poly Use N = 390 (Weighted %)	Other Patterns N = 790 (Weighted %)
Age						
12–14	1,670 (12.4)	1,187 (14.0)	302 (9.3)	61 (9.8)	53 (11.1)	67 (10.6)
15	3,257 (25.0)	2,255 (27.6)	669 (21.3)	95 (17.2)	65 (13.4)	173 (23.6)
16	3,389 (25.6)	2,109 (25.2)	830 (27.9)	158 (26.6)	91 (22.1)	201 (22.6)
17	2,900 (23.6)	1,733 (22.3)	698 (25.7)	148 (26.2)	98 (27.4)	223 (24.9)
≥18	1,491 (13.4)	805 (10.9)	384 (15.7)	103 (20.2)	81 (26.0)	118 (18.3)

Sex						
Female	6,464 (49.5)	4,127 (49.2)	1,621 (55.0)	222 (40.4)	119 (32.6)	375 (47.9)
Male	6,183 (50.5)	3,942 (50.8)	1,254 (45.0)	336 (59.6)	254 (67.4)	397 (52.1)

Race						
AI/AN/PH/HI	192 (1.0)	99 (0.8)	52 (1.0)	10 (1.8)	16 (2.9)	15 (1.2)
Asian	585 (5.2)	463 (6.2)	60 (2.1)	12 (1.9)	5 (1.2)	45 (11.0)
African American	1,836 (11.5)	1,446 (14.3)	252 (7.3)	51 (7.1)	26 (6.2)	61 (5.1)
White	6,377 (52.2)	3,664 (47.1)	1,695 (60.3)	346 (62.5)	215 (57.0)	457 (64.0)
Hispanic	945 (9.2)	664 (10.2)	198 (8.2)	31 (6.6)	18 (7.0)	34 (5.3)
Multi-Racial	2,464 (20.9)	1,584 (21.4)	583 (21.2)	104 (20.1)	92 (25.6)	101 (13.4)

Grade						
9	3,412 (26.8)	2,445 (30.4)	647 (21.6)	102 (18.2)	70 (16.5)	148 (21.2)
10	3,483 (25.7)	2,267 (26.3)	795 (24.5)	143 (24.6)	89 (19.6)	189 (27.5)
11	3,072 (24.1)	1,833 (22.8)	736 (26.9)	148 (25.4)	97 (25.1)	258 (24.4)
12	2,672 (23.4)	1,524 (20.4)	692 (27.0)	166 (31.8)	117 (38.8)	173 (26.9)

Sexual Orientation						
Heterosexual	10,217 (84.7)	6,531 (84.7)	2,353 (86.0)	441 (85.0)	280 (79.3)	612 (82.7)
Gay or Lesbian	330 (2.4)	203 (2.4)	75 (2.4)	13 (1.8)	16 (3.0)	23 (2.3)
Bisexual	1,068 (8.6)	635 (8.1)	257 (8.7)	59 (10.2)	42 (12.5)	75 (10.4)
Not sure	537 (4.3)	380 (4.8)	84 (2.9)	18 (3.1)	20 (5.2)	35 (4.6)

AI/AN/NH/PI American Indian/Alaskan Native/Native Hawaiian/Pacific Islander.

Other patterns include the patterns of tobacco use other than the four categories of interest. These include, sole, dual, and polyuse of other non-e-cigarette tobacco products.

**Fig. 1:**
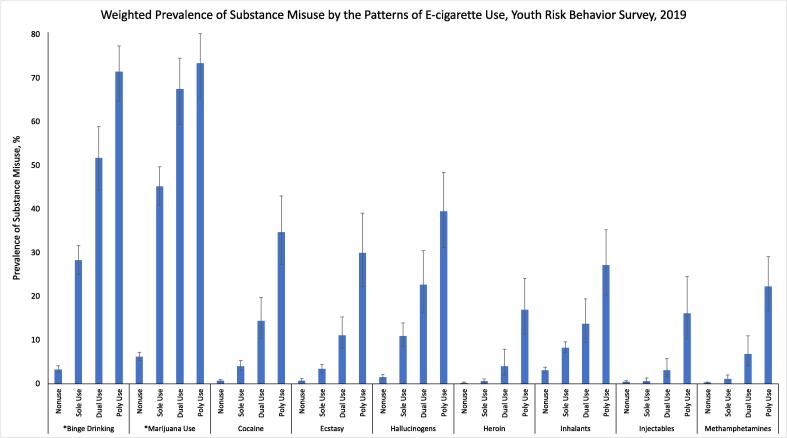
Weighted Prevalence of Substance Misuse by the Patterns of E-cigarette Use, Youth Risk Behavior Survey, 2019.

The prevalence of misuse for the substances studied varied, with past-30-day marijuana use being the highest (21.3%), followed by past-30-day binge drinking (13.5%), with the least being lifetime use of injectables (1.1%) (Table 2). Across all the substances explored in this study, the prevalence was highest among poly users, followed by dual users and then sole users (Table 2 and Fig. 1). For instance, the prevalence of lifetime cocaine use was 34.7% among poly users, 14.4% among dual users, 4.0% among sole users, and 0.7% among non-users.

**Table 2:** Prevalence of the Individual Substance Misuse Patterns by Patterns of E-cigarette Use, 2019 Youth Risk Behavior Surveillance System, United States.

Substance Misuse	Total Percent (95% CI)	Nonuse Percent (95% CI)	Sole Use Percent (95% CI)	Dual Use Percent (95% CI)	Poly Use Percent (95% CI)
Current Binge Drinking	13.5 (12.1–115.1)	3.2 (2.5–4.1)	28.3 (25.1–31.7)	51.7 (44.4–58.9)	71.5 (64.7–77.4)
Current Marijuana Use	21.3 (19.4–23.4)	6.2 (5.3–7.2)	45.2 (40.8–49.7)	67.5 (59.4–74.6)	73.4 (65.4–80.2)
Ever Use of Cocaine	3.4 (2.8–4.2)	0.7 (0.4–1.0)	4.0 (3.0–5.3)	14.4 (10.3–19.8)	34.7 (27.3–43.0)
Ever Use of Ecstasy	3.0 (2.5–3.6)	0.7 (0.5–1.2)	3.4 (2.6–4.4)	11.1 (8.0–15.3)	30.0 (22.3–39.1)
Ever Use of Hallucinogens	6.4 (5.3–7.8)	1.5 (1.1–2.1)	10.9 (8.5–13.9)	22.7 (16.4–30.5)	39.5 (31.2–48.4)
Ever Use of Heroin	1.3 (0.9–1.7)	0.2 (0.1–0.4)	0.6 (0.3–1.1)	4.0 (1.9–7.9)	16.9 (11.4–24.1)
Ever Use of Inhalants	5.9 (5.3–6.6)	3.1 (2.5–3.8)	8.2 (7.1–9.6)	13.7 (9.5–19.4)	27.2 (20.3–35.3)
Ever Use of Injectables	1.1 (0.8–1.7)	0.4 (0.2–0.7)	0.6 (0.3–1.3)	3.1 (1.6–5.8)	16.1 (10.2–24.6)
Ever Use of Methamphetamines	1.7 (1.3–2.1)	0.3 (0.1–0.4)	1.1 (0.6–2.0)	6.8 (4.1–11.0)	22.3 (16.7–29.1)

In multivariable analysis adjusting for age, sex, race/ethnicity, and sexual orientation, poly users, dual users, and sole users had a significantly higher prevalence of reporting substance misuse compared to non-users (Table 3). For example, sole users, dual users, and poly users had 8.0 (95% CI: 6.6–10.2) times, 14.7 (95% CI: 11.3–19.1) times, and 20.9 (95% CI: 16.2–26.9) times higher adjusted prevalence of reporting past-30-day binge drinking compared to non-users. This pattern was seen across all the explored substances (Table 3). Additional adjustment for depression, which tends to co-occur with tobacco and substance use, did not significantly alter the observed estimates. When sole e-cigarette users were further stratified by frequency of use, the estimates of the association between sole e-cigarette use and the high-risk substance use behaviors was higher for frequent users (≥20 days in past 30 days) than for less frequent users (<20 days in past 30 days) (Table 3). When using sole e-cigarette users as the reference, dual and poly users had significantly higher adjusted prevalence of all nine substances explored (Table 4).

**Table 3:** Prevalence Ratios of the Association Between Substance Misuse and E-cigarette-Specific Tobacco Use Patterns, 2019 Youth Risk Behavior Surveillance System, United States.

Substance Misuse	Model 1 aPR (95% CI)	Model 2 aPR (95% CI)
Current Binge Drinking		
Nonuse (N = 8,127)	Ref	Ref
Sole E-cigarette <20 days per month (N = 2,178) ≥20 days per month (N = 715)	8.0 (6.3–10.2)7.0 (5.5–8.9)10.9 (8.3–14.2)	7.8 (6.1–10.0)6.8 (5.3–8.7)10.5 (7.9–13.9)
Dual Use (N = 567)	14.7 (11.3–19.1)	14.3 (10.8–18.8)
Poly Use (N = 390)	20.9 (16.2–26.9)	19.7 (15.0–25.9)

Current Marijuana Use		
Nonuse	Ref	Ref
Sole E-cigarette <20 days per month ≥20 days per month	7.4 (6.2–8.8)6.5 (5.4–8.0)10.1 (8.6–11.8)	7.2 (6.0–8.5)6.3 (5.2–7.7)9.7 (8.3–11.3)
Dual Use	10.8 (9.2–12.8)	10.5 (8.9–12.4)
Poly Use	11.0 (9.1–13.4)	10.4 (8.6–12.6)

Ever Use of Cocaine		
Sole E-cigarette <20 days per month ≥20 days per month	6.8 (4.3–10.8)5.0 (3.1–8.2)12.7 (7.2–22.2)	6.2 (4.0–9.6)4.6 (2.8–7.6)10.8 (6.3–18.5)
Dual Use	22.8 (13.8–37.7)	20.5 (12.4–33.8)
Poly Use	50.1 (30.6–82.2)	43.0 (25.4–72.7)

Ever Use of Ecstasy		
Sole E-cigarette <20 days per month ≥20 days per month	4.8 (2.6–8.8)4.3 (2.3–8.2)7.0 (3.3–14.9)	4.5 (2.5–8.0)4.2 (2.2–7.7)6.2 (2.9–13.2)
Dual Use	15.7 (8.4–29.2)	14.9 (8.0–27.8)
Poly Use	36.8 (21.6–62.7)	33.0 (19.4–56.0)

Ever Use of Hallucinogens		
Sole E-cigarette <20 days per month ≥20 days per month	6.9 (4.9–9.7)5.2 (3.5–7.7)11.6 (8.0–16.9)	6.2 (4.4–8.7)4.7 (3.2–6.8)10.5 (7.3–15.1)
Dual Use	12.5 (9.0–17.4)	11.4 (8.2–15.9)
Poly Use	20.2 (14.0–29.0)	17.6 (12.5–24.9)

Ever Use of Heroin		
Sole E-cigarette <20 days per month ≥20 days per month	3.4 (1.3–8.8)3.7 (1.4–10.3)3.0 (0.8–11.7)	3.2 (1.2–8.2)3.3 (1.2–9.2)2.6 (0.7–10.4)
Dual Use	18.3 (7.7–43.4)	17.2 (6.8–43.3)
Poly Use	73.8 (35.8–152.2)	60.6 (28.1–130.7)

Ever Use of Inhalants		
Sole E-cigarette <20 days per month ≥20 days per month	2.8 (2.2–3.6)2.7 (2.1–3.6)3.0 (2.0–4.4)	2.4 (1.9–3.1)2.4 (1.8–3.1)2.5 (1.6–3.8)
Dual Use	4.4 (2.9–3.6)	3.8 (2.5–5.9)
Poly Use	9.3 (6.6–13.1)	7.4 (5.3–10.4)

Ever Use of Injectables		
Sole E-cigarette <20 days per month ≥20 days per month	1.8 (0.6–5.5)1.9 (0.6–6.4)1.5 (0.3–6.2)	1.6 (0.6–4.6)1.7 (0.5–5.3)1.3 (0.3–5.2)
Dual Use	9.3 (4.0–22.0)	8.3 (3.4–20.1)
Poly Use	39.0 (19.2–79.4)	29.5 (13.9–62.7)

Ever Use of Methamphetamines		
Sole E-cigarette <20 days per month ≥20 days per month	5.5 (2.2–14.0)4.7 (1.6–13.3)8.6 (3.1–23.8)	5.3 (2.1–13.4)4.5 (1.6–12.5)7.9 (2.8–22.4)
Dual Use	27.9 (13.0–60.1)	26.6 (12.0–59.4)
Poly Use	90.2 (49.5–164.4)	80.3 (41.7–154.5)

aPR, Adjusted prevalence ratio; CI, Confidence interval.

Model 1: Adjusted for age, sex, race/ethnicity, and sexual orientation.

Model 2: Model 1 + Depression as a proxy for mental health.

**Table 4:** Prevalence Ratios of the Association Between Substance Misuse and E-cigarette-Specific Tobacco Use Patterns Using Sole E-cigarette Use as Reference, 2019 Youth Risk Behavior Surveillance System, United States.

Substance Misuse	Sole E-cigarette	Dual Use	Poly Use
Current Binge Drinking	Ref	1.8 (1.6–2.1)	2.5 (2.1–3.0)
Current Marijuana Use	Ref	1.5 (1.3–1.7)	1.5 (1.3–1.7)
Ever Use of Cocaine	Ref	3.3 (2.2–4.9)	7.0 (4.6–10.6)
Ever Use of Ecstasy	Ref	3.3 (2.2–4.9)	7.3 (4.5–11.7)
Ever Use of Hallucinogens	Ref	1.8 (1.4–2.5)	2.9 (2.1–3.8)
Ever Use of Heroin	Ref	5.5 (2.3–13.2)	19.2 (9.1–40.6)
Ever Use of Inhalants	Ref	1.6 (1.1–2.2)	3.1 (2.3–4.2)
Ever Use of Injectables	Ref	5.2 (1.8–15.1)	18.3 (6.6–50.5)
Ever Use of Methamphetamines	Ref	5.0 (2.1–11.7)	15.1 (6.8–33.3)

Adjusted for age, sex, race/ethnicity, sexual orientation, and depression.

## 4 Discussion

Using a large, nationally representative sample of high school students in the US, we found that the patterns of tobacco use among youth are variable with sole e-cigarette use being the most common pattern of use. Additionally, there was a higher prevalence of substance misuse among youth who reported dual use and poly use than those who reported nonuse or sole use of e-cigarettes.

Similar to recent reports, we found that although sole use of e-cigarettes was the most common pattern of tobacco use among youth, the use of multiple tobacco products was not uncommon. (Park-Lee et al., 2022, Jebai et al., 2022) Evidence from the 2022 National Youth Tobacco survey showed that about 5% of high school students in the US reported using two or more tobacco products in the past 30 days. (Park-Lee et al., 2022) In another study, Jebai et al. showed that there was a significant increase in the prevalence of dual use of e-cigarettes and cigarettes as well as poly tobacco use among US youth between 2011 and 2020. (Jebai et al., 2022) This is a concerning pattern of tobacco use among youth since the effects of multiple tobacco product use include greater exposure to nicotine and an increased likelihood of addiction. (Gomez et al., 2020) Furthermore, the use of multiple nicotine-containing products during adolescents is concerning as the effects of nicotine exposure on developing brain function and behavior can be long-lasting due to interference with the brain's reward network. (Yuan et al., 2015, Vanyukov et al., 2012, Goriounova and Mansvelder, 2012a) There have also been reports of the increased risk of impulsivity, irritability, and anxiety among adolescents who report the use of multiple tobacco products. (Gomez et al., 2020, Miller and Hurd, 2017).

In addition to the health effects that are associated with multiple tobacco product use, our findings suggest that such patterns of tobacco use are associated with other high-risk substance use behaviors, which may further increase the health risks associated with tobacco use. In a study assessing adolescent risk behavior and use of e-cigarettes and combustible cigarettes, participants who reported sole e-cigarette use were 3.7 times (PR: 3.49; 95% CI: 3.16–4.32) more likely to report marijuana use in the past 30-days, while dual users of e-cigarettes and combustible cigarettes were 5.2 times (PR: 5.22; 95% CI: 4.42–6.18) more likely to report such high-risk behavior compared to nonusers. (Demissie et al., 2017) The aforementioned study also showed that dual use was also significantly associated with high-risk sexual behaviors such as having four or more lifetime sexual patterns among high school students. We extended these findings to examine the association of the patterns of e-cigarette use, including poly use, and a broad range of substances of abuse. Additionally, we examined if dual and poly users were different from sole users. We found that participants who reported dual or poly use were significantly more likely to engage in substance misuse than participants who reported sole e-cigarette use.

The coexistence of tobacco use and substance misuse, particularly among youth who use multiple tobacco products, may be explained by the gateway hypothesis or the common liability theory. The gateway hypothesis states that legal substances which include nicotine and alcohol may precede the use of illicit substances such as methamphetamine and cocaine. (Lozano et al., 2021) Thus, youth who use tobacco products may subsequently misuse other substances. However, due to the cross-sectional nature of our study, we cannot determine the directionality of the observed associations. For instance, while studies have shown that tobacco use can lead to initiation of alcohol consumption, the reverse is also true. (Lynskey et al., 1998) Therefore, preventing alcohol misuse among youth may also help curb tobacco use initiation. The common liability theory might also explain the association between tobacco use and substance misuse in adolescence. (Goriounova and Mansvelder, 2012a) During adolescence, the growing brain undergoes structural and functional changes that predispose adolescents to risk taking behaviors such as the use of tobacco and illicit substances. (Yuan et al., 2015) The observed clustering between multiple tobacco product use and substance misuse may thus be explained by their shared risk factor such as rejection of social norms, peer influence, and emotional and behavioral problems. (Demissie et al., 2017, Goriounova and Mansvelder, 2012a, Ozer et al., 2011).

Our findings highlight the high prevalence of dual and poly tobacco use among youth and the clustering of such patterns of tobacco use with high-risk substance use behaviors. The high prevalence of tobacco use among this age group emphasizes the need for stricter enforcement of policies that restrict youth access to tobacco products such as the Tobacco-21 legislation. Additionally, policies such as the e-cigarette flavor ban, which may reduce the appeal of e-cigarette use among youth, must be strictly enforced. Our findings also highlight the importance of comprehensive approaches to addressing youth tobacco use that include screening for high-risk substance use and counseling youth against these behaviors. Since e-cigarette use, particularly, when used with other tobacco products clusters with high-risk substance misuse, it can serve as a marker for identifying youth who may be misusing substances of abuse. Stricter enforcement of policies that restrict youth access to tobacco products and illicit substances are needed. Additionally, healthcare providers have a role to play in curbing youth tobacco and high-risk substance use. A prior study showed that brief counsel by healthcare providers can mitigate adolescent risk behaviors. (Boakye et al., 2023) Despite this evidence, the rate of tobacco screening among youth in the US by healthcare providers has been reported to be low.yyy^32^ To tackle tobacco use and substance misuse among youth, concerted efforts that include policies to restrict access, education and counseling on the health effects, as well as availability of evidence-based age-appropriate cessation interventions are needed.

## 5 Limitations

The findings of our study should be cautiously interpreted, due to some limitations. The data used in this study were self-reported with the potential for recall bias and misclassification. Additionally, considering the cross-sectional nature of this study, we cannot determine the directionality of these associations. Furthermore, we did not have information on factors such as susceptibility to use, sensation-seeking, and social support, which may influence the observed associations; thus, there is the potential for residual confounding. Also, some of the prevalence ratios have wide confidence intervals, indicating lowered precision in the presented effect sizes. Lastly, the YRBSS survey is representative of only school-going adolescents and is therefore not representative of non-school going adolescents who may have higher rates of tobacco use and substance misuse than those enrolled in schools.

## 6 Conclusions

Sole use of e-cigarette, as well as the concurrent use with other tobacco products is associated with substance misuse among adolescents. Furthermore, individuals who reported dual and poly use were significantly more likely to engage in substance misuse than individuals who reported exclusive e-cigarette use. These findings highlight the clustering of tobacco use with substance misuse and emphasize the need for comprehensive efforts to address health-risk behaviors, including tobacco prevention measures that also address high-risk substance use among adolescents.

## CRediT authorship contribution statement

**John Erhabor:** Conceptualization, Methodology, Formal analysis, Writing – original draft. **Ellen Boakye:** Conceptualization, Methodology, Writing – review & editing. **Ngozi Osuji:** Writing – review & editing. **Olufunmilayo Obisesan:** Writing – review & editing. **Albert D. Osei:** Writing – review & editing. **Hassan Mirbolouk:** Writing – review & editing. **Andrew C. Stokes:** Writing – review & editing. **Omar Dzaye:** Writing – review & editing. **Omar El-Shahawy:** Writing – review & editing. **Carlos J. Rodriguez:** Writing – review & editing. **Glenn A. Hirsch:** Writing – review & editing, Supervision. **Emelia J. Benjamin:** Writing – review & editing, Supervision. **Andrew P. DeFilippis:** Writing – review & editing. **Rose Marie Robertson:** Conceptualization, Writing – review & editing, Supervision, Funding acquisition. **Aruni Bhatnagar:** Conceptualization, Writing – review & editing, Supervision, Funding acquisition. **Michael J. Blaha:** Conceptualization, Methodology, Writing – review & editing, Supervision, Funding acquisition.

## Declaration of Competing Interest

The authors declare that they have no known competing financial interests or personal relationships that could have appeared to influence the work reported in this paper.

## Data Availability

Data used is publicly available.
