# Supplementary material for: Patterns of tobacco product use and substance misuse among adolescents in the United States
Source: Prev Med Rep. 2023 Apr 15;33:102207. doi: 10.1016/j.pmedr.2023.102207 (PMC10201858; doi:10.1016/j.pmedr.2023.102207)
Supplement: Supplementary data 1 [file mmc1.docx]

Supplementary Table S1: The exact Questions Used to Assess the Nine Substances of Abuse, 2019 Youth Risk Behavior Surveillance System, United States.

| Substance | Question |
| --- | --- |
| Current Binge Drinking | During the past 30 days, on how many days did you have 4 or more drinks of alcohol in a row, that is, within a couple of hours (if you are female) or 5 or more drinks of alcohol in a row, that is, within a couple of hours (if you are male)? |
| Current Marijuana Use | During the past 30 days, how many days did you use marijuana? |
| Ever Use of Cocaine | During your life, how many times have you used any form of cocaine, including powder, crack, or freebase? |
| Ever Use of Ecstasy | During your life, how many times have you used ecstasy (also called MDMA)? |
| Ever Use of Hallucinogens | During your life, how many times have you used hallucinogenic drugs, such as LSD, acid, PCP, angel dust, mescaline, or mushrooms? |
| Ever Use of Heroin | During your life, how many times have you used heroin (also called smack, junk, or China White? |
| Ever Use of Inhalants | During your life, how many times have you sniffed glue, breathed the contents of aerosol spray can or inhaled any paints or spray to get high? |
| Ever Use of Injectables | During your life, how many times have you used needle to inject any illegal in your body? |
| Ever Use of Methamphetamines | During your life, how many times have you used methamphetamines (also called speed, crystal met, crank, ice or meth)? |
| Response of having used a specific substance even once within the duration specified was regarded as use of that substance | |
